# Machine Learning-Enabled Optimization of Interstitial Fluid Collection via a Sweeping Microneedle Design

**DOI:** 10.1021/acsomega.3c01744

**Published:** 2023-05-31

**Authors:** Ceren Tarar, Erdal Aydın, Ali K. Yetisen, Savas Tasoglu

**Affiliations:** † Department of Biomedical Sciences and Engineering, Koç University, Sariyer, Istanbul 34450, Turkey; ‡ Department of Chemical and Biological Engineering, Koç University, Sariyer, Istanbul 34450, Turkey; § TUPRAS Energy Center (KUTEM), Koç University, Istanbul 34450, Turkey; ∥ Department of Chemical Engineering, Imperial College London, London SW7 2AZ, U.K.; ⊥ Koc University Is Bank Artificial Intelligence Lab (KUIS AILab), Koç University, Sariyer, Istanbul 34450, Turkey; # Koç University Translational Medicine Research Center (KUTTAM), Koç University, Istanbul 34450, Turkey; ∇ Boğaziçi Institute of Biomedical Engineering, Boğaziçi University, Çengelköy, Istanbul 34684, Turkey; ○ Department of Mechanical Engineering, Koç University, Sariyer, Istanbul 34450, Turkey; ◆ Koç University Arçelik Research Center for Creative Industries (KUAR), Koç University, Sariyer, Istanbul 34450, Turkey; ¶ Physical Intelligence Department, Max Planck Institute for Intelligent Systems, 70569 Stuttgart, Germany

## Abstract

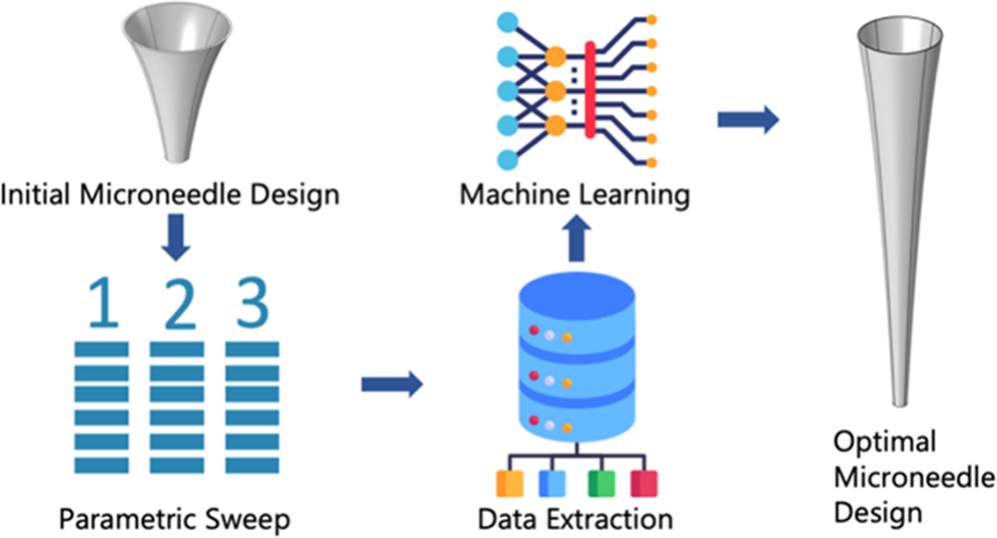

Microneedles (MNs) allow for biological fluid sampling and drug delivery toward the development of minimally invasive diagnostics and treatment in medicine. MNs have been fabricated based on empirical data such as mechanical testing, and their physical parameters have been optimized through the trial-and-error method. While these methods showed adequate results, the performance of MNs can be enhanced by analyzing a large data set of parameters and their respective performance using artificial intelligence. In this study, finite element methods (FEMs) and machine learning (ML) models were integrated to determine the optimal physical parameters for a MN design in order to maximize the amount of collected fluid. The fluid behavior in a MN patch is simulated with several different physical and geometrical parameters using FEM, and the resulting data set is used as the input for ML algorithms including multiple linear regression, random forest regression, support vector regression, and neural networks. Decision tree regression (DTR) yielded the best prediction of optimal parameters. ML modeling methods can be utilized to optimize the geometrical design parameters of MNs in wearable devices for application in point-of-care diagnostics and targeted drug delivery.

## 1 Introduction

Microneedles (MNs) are microscale hypodermic hollow tubes that are used for minimally invasive drug delivery and biological fluid sampling, aiming at ensuring a painless patient experience.^1^ MNs have several significant advantages compared to traditional methods of fluid sampling.^2^ The most important ones are controlled drug release over time, cost efficiency, and mobility. While traditional hypodermic injections need to be performed by a medical specialist, MN patches can easily be applied by the patient. Moreover, MNs enable direct access to the skin whereas hypodermic injections penetrate the muscle where the immune response is weaker.^3^ Various MN designs are proposed in the literature^4^ in various shapes and with heights varying in the range of 25–2000 μm.^5^ Design optimization methods include computational fluid dynamics (CFD), mathematical modeling, and experimental methods such as mechanical testing.

Machine learning (ML) is a method of advanced data analysis where mathematical models are generated based on a data set to determine the behavior of a system for any given set of inputs. ML algorithms are exclusively based on previous outcomes, thus not affected by external factors, which ensures unbiased predictions. Furthermore, the algorithms can process large sets of data in a short period of time and provide results tremendously faster than the human brain. Utilizing ML in industrial applications has gained momentum throughout the last decade, particularly to solve engineering problems without the need for high-cost technologies and setups.^6^ Likewise, the potential of using ML for physical sciences has also emerged.^7^ A specific use of ML is in optimization problems, where a model can define the optimal parameters for a system, considering pre-defined performance metrics and boundary conditions.^8^

**Figure 1:**
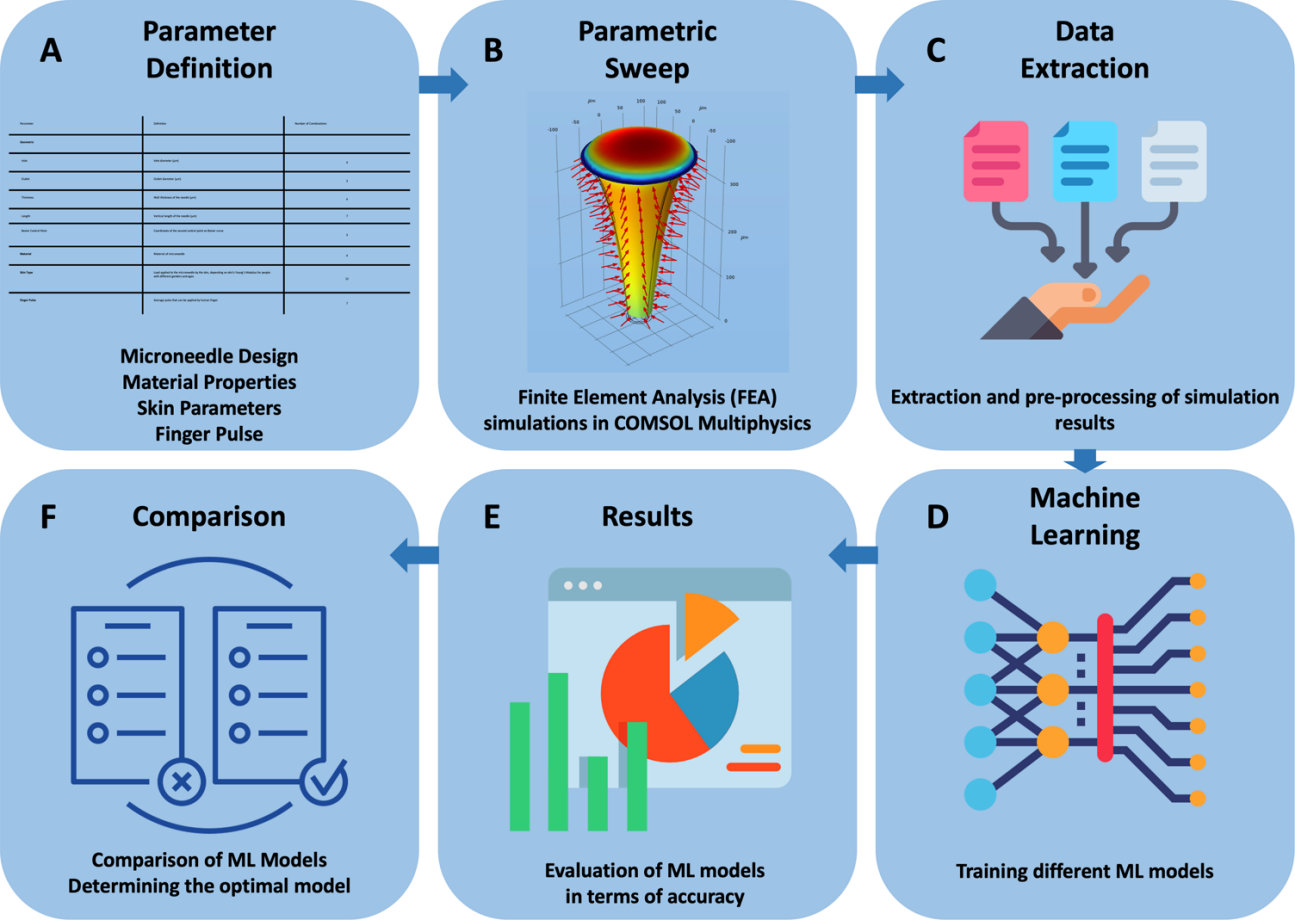
Workflow of the model development. (A) Definition of MN parameters that affect overall performance. (B) Stationary parametric sweep simulation in COMSOL Multiphysics with Laminar Flow and Solid Mechanics modules including all combinations of the defined parameters. (C) Exporting parameter combinations and the corresponding volumetric flow rates (VFRs) as a data set. (D) Training machine learning (ML) models with a subset of the data. (E) Evaluating overall performance of ML models in terms of mean squared error (MSE) and R-squared score (R-2). (F) Comparison of ML models with respect to their performance metrics.

In this study, the finite element method (FEM) and ML algorithms have been integrated to optimize the geometrical and physical parameters of a microneedle in order to determine a design within the fabrication limitations. The ML-designed MN can wick the highest amount of interstitial fluid (ISF) in one insertion. Finite element analyses are performed in COMSOL Multiphysics. Parameters that affect the amount of collected ISF were determined, and a time-dependent parametric sweep is performed to obtain a data set. ML algorithms were created in a MATLAB computing environment using its own multi-paradigm programming language. Various algorithms including linear regression, decision tree regression, support vector regression, and neural networks were performed and compared in terms of accuracy. This work, to the best of author’s knowledge, presents the first simulation-based ML framework to optimize MN design in terms of geometrical parameters. The framework is customizable, allowing for the optimization of the design for various materials, skin types, and shapes (Figure 1).

## 2 Methods

### 2.1 Design of Microneedle Surfaces

**Figure 2:**
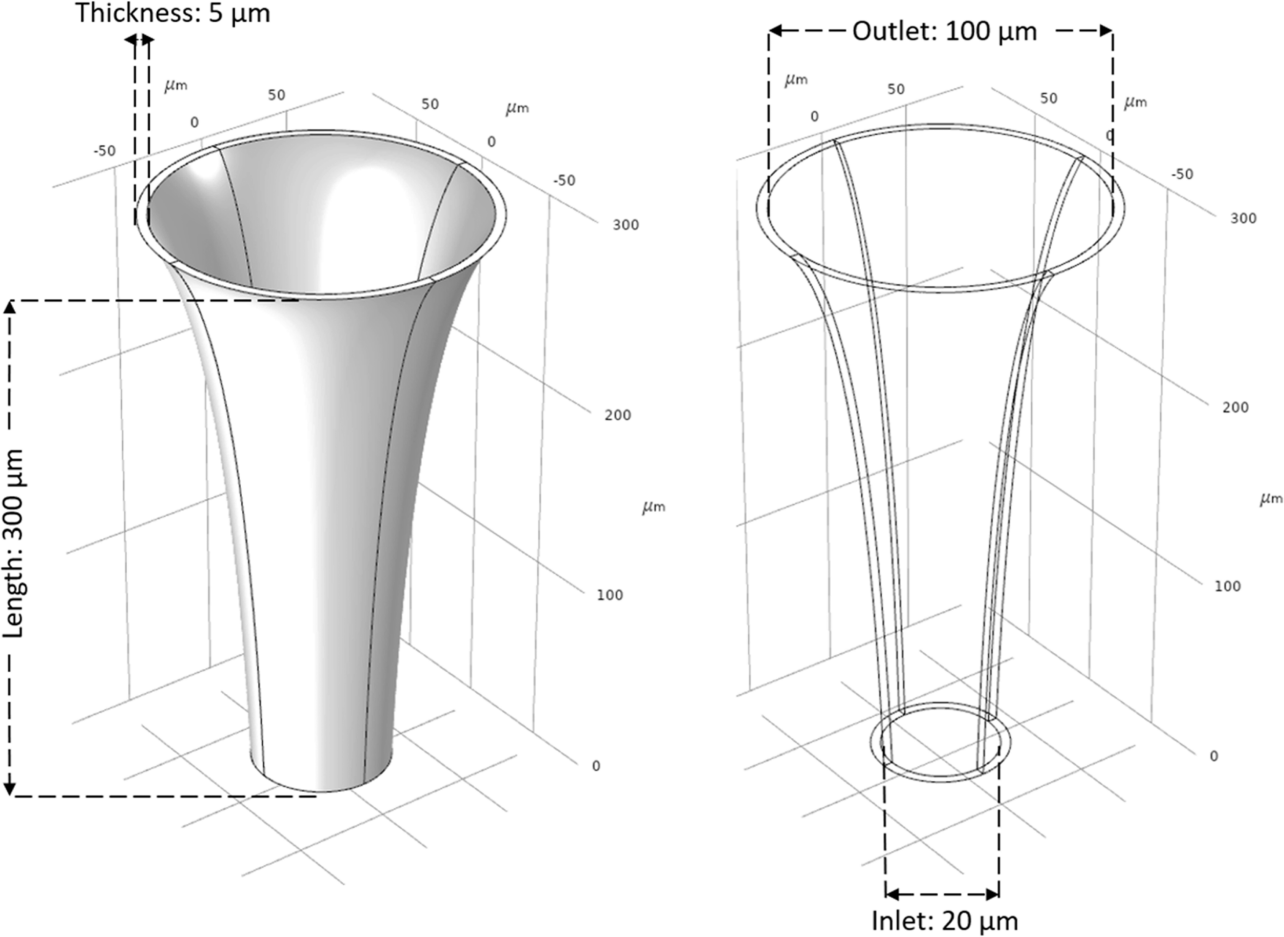
Initial design of the MN. A 2D sketch is drawn as the cross-sectional profile of the MN which consists of two line segments that define the inlet and outlet diameters, and a QBC that defines the curvature of the MN. The 2D sketch is revolved 360° around the *z*-axis in order to create the 3D model of the MN.

A single default design for a microneedle was created in a COMSOL environment with the dimensions shown in Figure 2. The initial microneedle had a length of 350 μm, an inlet diameter of 20 μm, an outlet diameter of 100 μm, and a wall thickness of 5 μm. The MN had a concave profile, which was defined by a quadratic Bezier curve (QBC). QBC that determines the inner surface of the microneedle was defined with three Cartesian control points ((20, 0, 0), (50, 0, 233), (100, 0, 350) [μm]) with equal weights. To obtain a 3D model, 2D cross-section of the microneedle as a closed curve with two line segments and two QBCs revolved with reference to the *z*-axis using the COMSOL Revolve function.

**Figure 3:**
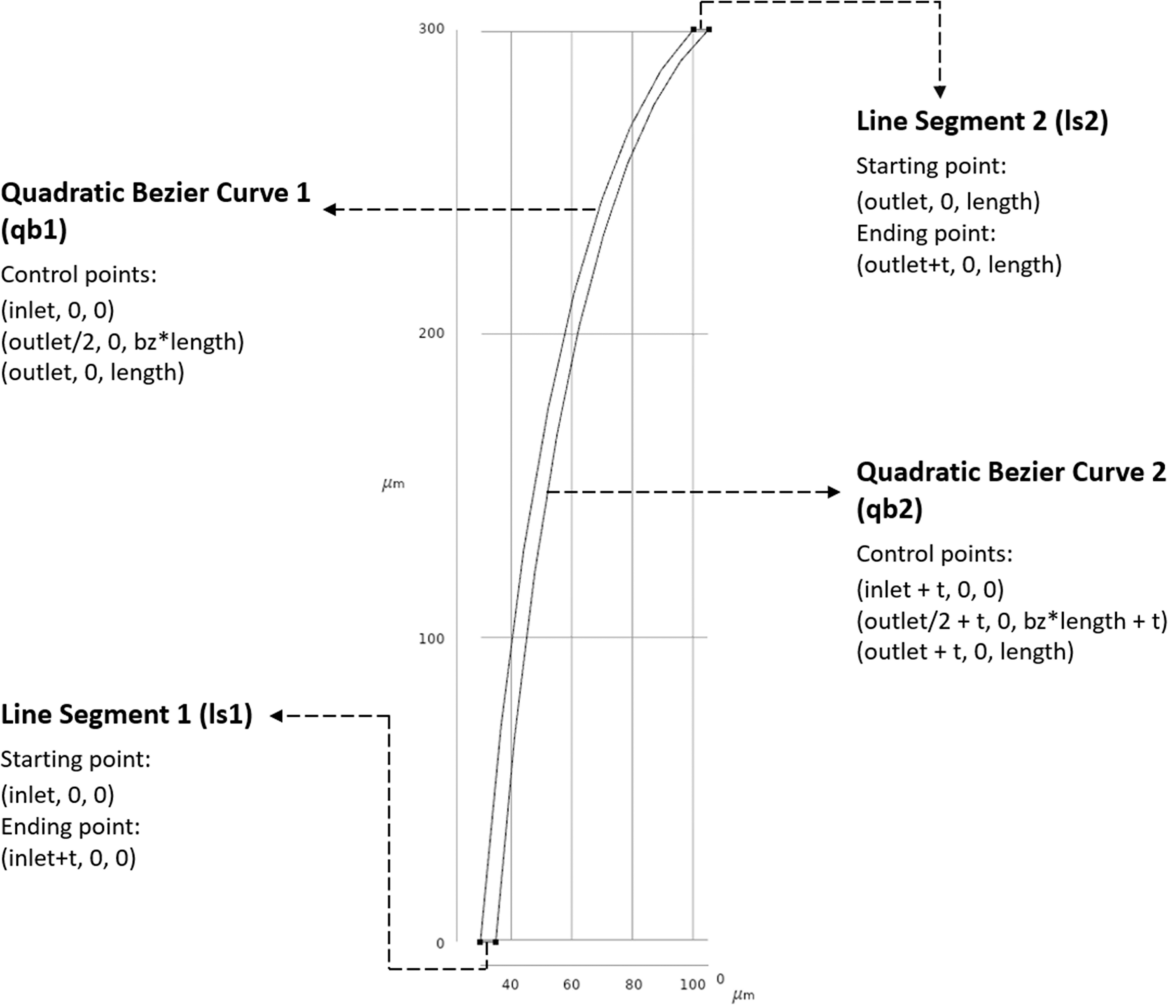
2D profile of the MN below 360° revolving around the *z*-axis. Line segments and curves are indicated with their corresponding reference coordinates. Line segments that define inlet and outlet diameters are indicated with their starting and ending points, QBCs are defined with their control points. Each control point in a QBC has equal weight. All coordinates are in (*x*,*y*,*z*) format.

Initial geometrical parameters was based on previous designs in the literature.^9,10^ Similarly, the range of parameters were defined within the fabrication limitations for designated materials of the MN. Table 1 shows all the parameters of the simulation. Table 2 shows the range of geometrical parameters used for the parametric sweep. The range and steps of the variables were selected considering optimal conditions for ML models, as well as fabrication limitations. The outlet diameter (outlet) values were defined to be larger than the respective inlet diameter (inlet) at all combinations. The minimum wall thickness was defined as 3 μm and the maximum wall thickness was 7 μm. Bezier control point (bz) was defined as shown in Figure 3. MNs with 1008 different shapes were evaluated in total.

**Table 1:** Parameters Defining the Geometry of a Single Microneedle and Number of Variations Used in COMSOL Multiphysics

parameter	definition	number of combinations
geometric		
inlet	inlet diameter (μm)	4
outlet	outlet diameter (μm)	3
thickness	wall thickness of the needle (μm)	4
length	vertical length of the needle (μm)	7
Bezier control point	coordinates of the second control point on the Bezier curve	3
material	material of the microneedle	4
skin type	load applied to the microneedle by the skin, depending on skin’s Young’s modulus for people of different genders and ages	12

**Table 2:** Determined Values of Each Geometrical Parameter

geometrical parameters	values
inlet diameter [μm] (inlet)	10, 20, 30, 40
outlet diameter [μm] (outlet)	80, 100, 120
Bezier control point (bz)	0.7, 0.8, 0.9
wall thickness [μm] (t)	3, 5, 7, 9
length [μm] (len)	100, 300, 500, 700, 900, 1100, 1300

### 2.2 COMSOL Simulations

Mechanical CFD simulations were performed in COMSOL Multiphysics. A method was created to design the optimal MN for ISF collection, and the fluid parameters were defined. Reynold’s Number for the flow in a single MN is defined as
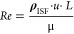
1where **ρ**_ISF_ is the density of ISF (1000 kg m^–3^),^11^*u* is the flow velocity (0.001 m s^–1^), the maximum reported velocity, *L* is the length of the MN (1300 μm), and μ is the dynamic viscosity of ISF (3.5 × 10^–3^ kg m^–1^·s^–1^).^11^ Length is assumed to be maximum, with the resulting maximum Reynold’s number of 10. Thus, the flow is assumed to be laminar.

Skin types are categorized according to age and gender, with intervals of 10 years. The load applied to the MN by the skin is defined as
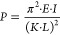
2where *P* is the applied load, *E* is Young’s Modulus, *I* is the second moment of area of the cross-section of the needle, *L* is the length of the needle, and *K* is the effective length factor.^12^Table 3 shows skin types and their corresponding Young’s moduli^13^ and applied loads.

**Table 3:** Skin Types, Their Corresponding Average Young’s Moduli and Loads Applied to the MN by the Skin

skin type	age	gender	Young’s modulus [MPa]	applied load [N]
M_20-30	20–30	male	250	1.47 × 10^–3^
M_30-40	30–40	male	200	1.17 × 10^–3^
M_40-50	40–50	male	200	1.17 × 10^–3^
M_50-60	50–60	male	240	1.41 × 10^–3^
M_60-70	60–70	male	350	2.06 × 10^–3^
M-70-80	70–80	male	500	2.94 × 10^–3^
F_20-30	20–30	female	180	1.06 × 10^–3^
F_30-40	30–40	female	100	5.87 × 10^–3^
F_40-50	40–50	female	180	1.06 × 10^–3^
F_50-60	50–60	female	300	1.76 × 10^–3^
F_60-70	60–70	female	500	2.94 × 10^–3^
F_70-80	70–80	female	800	4.70 × 10^–3^

ISF pressure is within the range of −0.5 and −8.0 mmHg,^14^ where the negative sign refers to the “dehydrated state” within the lymph flow.^15^ ISF pressure is assumed to be 4.0 mmHg (∼530 Pa) and extraction pressure is assumed to be 4500 Pa based on the average human finger pulse.^10^ Bernuolli’s incompressible flow principle states that the sum of flow work, kinetic energy, and potential energy of the fluid remains constant throughout a rigid channel. Bernuolli’s equation can be implemented as
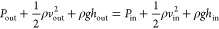
3where *P* is the pressure, *v* is the flow velocity, *g* is the gravitational acceleration, *h* is the height of elevation, and ρ is the fluid mass density for the outlet and any point on the *z*-axis. Assuming mass is conserved through the MN, (*A*_out_·*v*_out_ = *A*_in_·*v*_in_), the volumetric flow rate (VFR) is defined by the cross-sectional area multiplied by flow velocity at any point in the *z*-axis.
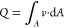
4Equation 4 defines VFR as a function of *r* throughout the MN, where *A* is the cross-sectional area, which is a function of the radius, and *v* is the velocity of individual points on the *z*-axis.

Simulations were performed using three different materials: stainless steel, titanium, and nickel–iron based on previous data in the literature.^16^

In COMSOL simulations, the mesh is generated based on the geometry of the model being simulated. The mesh is created by dividing the geometry into small, finite elements or cells, which are then used to discretize the problem and solve it numerically. In this simulation, the mesh is based on the MN geometry using a physics-controlled mesh and the mesh size is chosen as extra fine in order to accurately capture the features of interest in the geometry within the computational load limitations.

Inlet and outlet boundary conditions are specified in terms of pressure generated by an average human finger pulse. Gravity is defined as a constant toward the negative direction in the *z*-axis, and no-slip condition is assumed for all the materials regarding wall boundary conditions. All materials are assumed to be uncoated. Simulations are performed for room temperature and heat transfer is neglected.

A total of 48 384 combinations were simulated in COMSOL. Each simulation was designed as a two-step study, consisting of solid mechanics and fluid flow modules. At the first step, mechanical loads were applied to the MN and deformation was observed. In the second step, deformed MN properties were defined as the input, and fluid flow was evaluated in terms of the volumetric flow rate (VFR) [m^3^ s^–1^]. All data were exported as the main data set.

### 2.3 Optimization

In the design optimization process, an objective function that mathematically defines the problem was minimized considering equality and inequality constraints. Design optimization holds great value in biomedical applications, where the overall performance of an instrument can be improved significantly by maximizing factors such as efficiency, utilization, and durability, and minimizing cost and energy loss. While a theoretical relationship between geometrical parameters of a design and output value can be expressed as linear, empirical data rarely shows linearity. Nonlinear programming (NLP) refers to the optimization of a problem defined by nonlinear objective functions or constraints. The nonlinear maximization problem is mathematically defined as
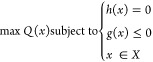
5where *Q*(*x*) is the objective function, *X* is a subset of *R*^5^ (array of geometrical parameters), and *h*(*x*) and *g*(*x*) are equality and inequality constraints, respectively. Upper and lower bounds of each parameter define inequality constraints. Equality constraints are defined by hyperparameters of the ML models.

### 2.4 Machine Learning

Each data point in the main data set represented a microneedle of a distinct size, shape, and material, and the respective VFR of ISF. The inlet diameter, outlet diameter, wall thickness, vertical length, and Bezier control point coefficients were determined to be the independent variables of the optimization problem while VFR was the single dependent output variable. Skin type, material, and finger pulse were treated as constants mainly because these parameters were introduced prior to the problem and did not need to be optimized. Features (independent variables) were normalized within the range −1 and 1, while target (dependent variable) was normalized within the range of 0 and 1. The data set was split randomly into training and testing data sets (random 80% of the data points were chosen as the training data set, and the remaining 20% constituted the testing data set). ML methods were implemented to pretrained models that were included in the MATLAB environment. ML methods aim to generate an objective function from discrete data points, so that for any given input, even if the input is unfamiliar to the model, a prediction of the output can be obtained. While almost none of the ML methods provide 100% accuracy, the most important objective of ML is to minimize the difference between actual output values and predicted output values, defined with mean squared error (MSE).

Linear regression (LR), regression ensemble (RE), decision tree regression (DTR), support vector regression (SVR), generalized additive model (GAM), Gaussian process regression (GPR), and neural network regression (NNR) were performed on the data. All the algorithms except for LR were also subjected to hyperparameter optimization. Hyperparameters were defined as the parameters that determined the behavior of the learning algorithm, whereas model parameters determined the behavior of the system. Hyperparameters of the algorithms were determined with Bayesian optimization, where a prior assumption over the possible objective functions was defined and as data were observed, the model was enhanced via Bayesian posterior updating.^17^ Hyperparameters to be tuned for each ML method were chosen automatically within *bayesopt* function in MATLAB, depending on the optimizable variables of the method. Hyperparameter optimization and learning processes were repeated for 30 iterations to verify the results over different distributions of training and testing data, and MSE values were reported for each method.

#### 2.4.1 Linear Regression (LR)

LR allows for fitting a straight line over the data (eq 6)
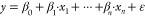
6where *y* is the dependent variable, *x*_*i*_ is the independent variable, β_*i*_ is the learning coefficients, and ε is the error.^18^ Optimal line was determined considering minimum MSE. LR is considered one of the simpler methods for regression due to low computation time and interpretable learning coefficients. Although LR can be useful for certain types of problems, real-world problems are usually defined by nonlinear relationships. Thus, using LR in nonlinear objective functions can result in oversimplification, meaning it cannot resolve complex patterns.^19^

#### 2.4.2 Regression Ensemble (RE)

An ensemble learning method is defined as the integration of a set of individually trained models where the predictions of these models are combined to determine the output. RE aims to strengthen the prediction by factoring in weak learners. Bootstrap aggregating (bagging)^20^ and boosting^21^ are the most common meta-algorithms used for ensemble learning.^22^ For bagging, pre-defined number of new data sets were generated from the original data by sampling from data uniformly. Regression was performed on each of these new data sets, creating individual models. The final output was determined by averaging the outputs. The same workflow was applied to boosting, but the data set generation was iterative.

#### 2.4.3 Decision Tree Regression (DTR)

DTR is a modification of decision tree modeling, where the training data set is segmented in the form of a tree based on a set of rules.^23^ Branches of the tree represent the decision criteria while nodes represent the subsection of data. DTR presents various advantages over similar learning algorithms including separate examination of predictor variables on target variables, analysis of complex data, and assessment of subsistent uncertainties.^24^ Although it has its advantages, DTR usually provides local optimal solution rather than the global optimal and is prone to sampling error.^19^

#### 2.4.4 Support Vector Regression (SVR)

SVR is an implementation of another supervised learning model, support vector machine (SVM). For a data set with *N* features, the objective of SVR is to find a hyperplane in an *N*-dimensional space that fits the maximum number of data points. An ε-sensitive region around the function termed ε-tube is introduced to the model in order to generalize SVM to SVR and the hyperplane is represented with support vectors defined by samples outside the boundary of ε-tube.^25^ The main advantage of SVR is the capacity to define the acceptable error margin, which enables tuning over tolerance. Previous studies have shown that for smaller data sets, SVR can be an alternative to neural networks.^26^

#### 2.4.5 Generalized Additive Model (GAM)

GAM is an interpretation of LR, where constant coefficients defined in a linear model are defined by smooth functions called splines, enabling the objective function itself to learn nonlinear features. GAM theory presents a flexible method for identifying nonlinear covariate effects in likelihood-based regression models.^27^ GAM is incredibly useful for upgrading existing LR models, although like LR, overfitting can be an issue with GAMs, especially without cross-validation. GAMs are also prone to error if a large number of smoothing parameters are involved. Yet, disadvantages can be minimized by the selection of appropriate properties such as the signal to-noise ratio, type of response, or number of covariates.^28^

#### 2.4.6 Gaussian Process Regression (GPR)

The Gaussian process is a collection of a finite number of random variables that have joint Gaussian distributions.^29^ GPR is based on Gaussian (normal) distribution, where a GPR model extracts a prior model to limit the possible forms of the unknown function and then updates this prior model to generate a posterior GPR as the final functional model. This process allows for inferring an unknown functional relationship from a training data set.^30^ Previous studies have shown high-performance results with GPR models for predictive modeling, although missing input parameters had a negative effect on their performance.^31^

#### 2.4.7 Neural Network Regression (NNR)

The architecture of NNR is based on biological neural systems. As such, nodes represent neurons and connections represent synapses. Moreover, current studies on neural networks (NNs) are mainly focused on gaining a deeper understanding of human learning processes and implementing these to ML methods.^32^ Artificial NNs offer numerous advantages including missing input tolerance, fault tolerance, gradual corruption, and parallel processing capability. This method is also highly dependent on the hardware, has unmanageable amount of network structure variations, and it can be difficult to examine the behavior of the learning process.^33^

## 3 Results and Discussion

### 3.1 COMSOL Simulation Results

**Figure 4:**
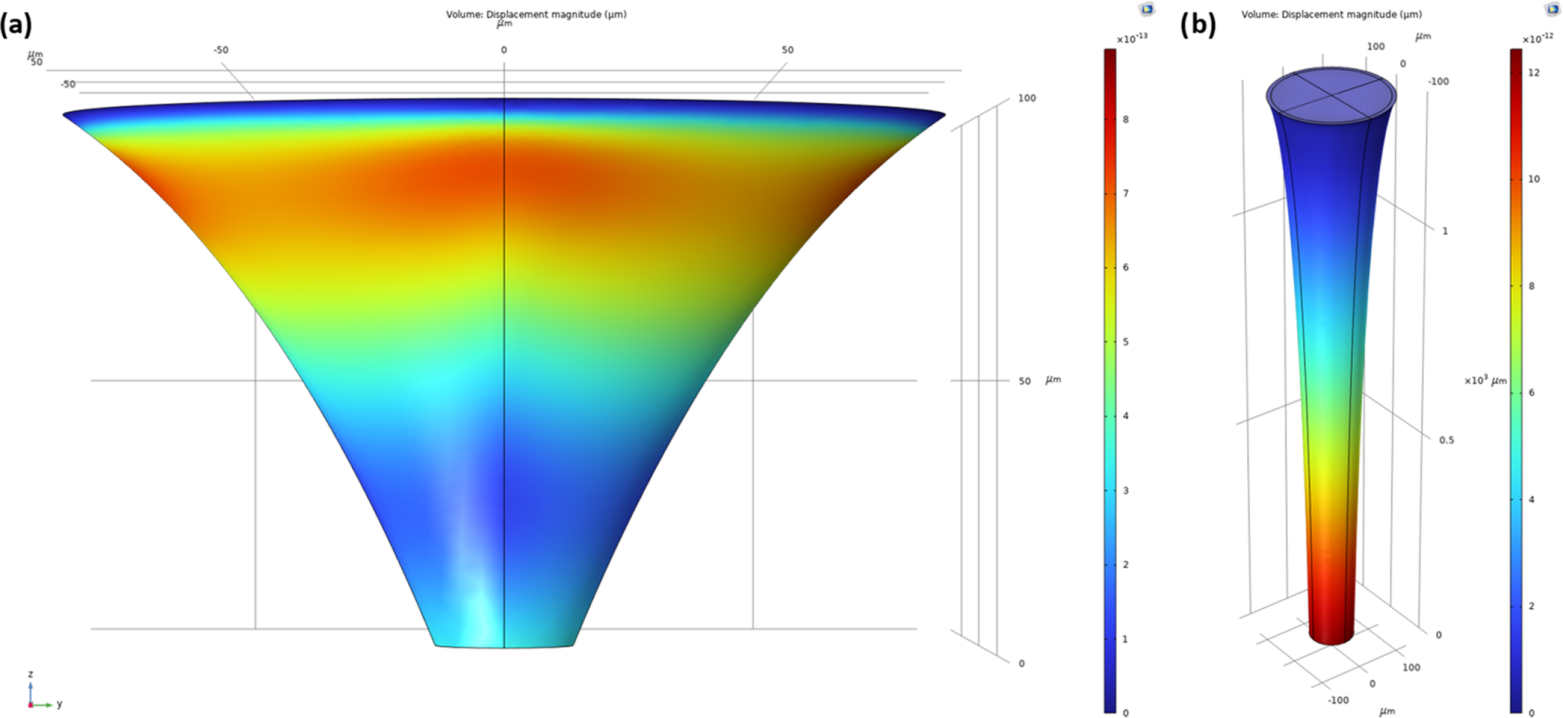
Displacement magnitudes shown in the COMSOL environment for geometrical parameters at their (a) minima and (b) maxima. Skin type is M_20-30 (male, aged between 20 and 30) and MN material is stainless steel. Average volumetric flow rates are 1.026 × 10^9^ and 4.213 × 10^9^ μm^3^ s^–1^, respectively.

**Figure 5:**
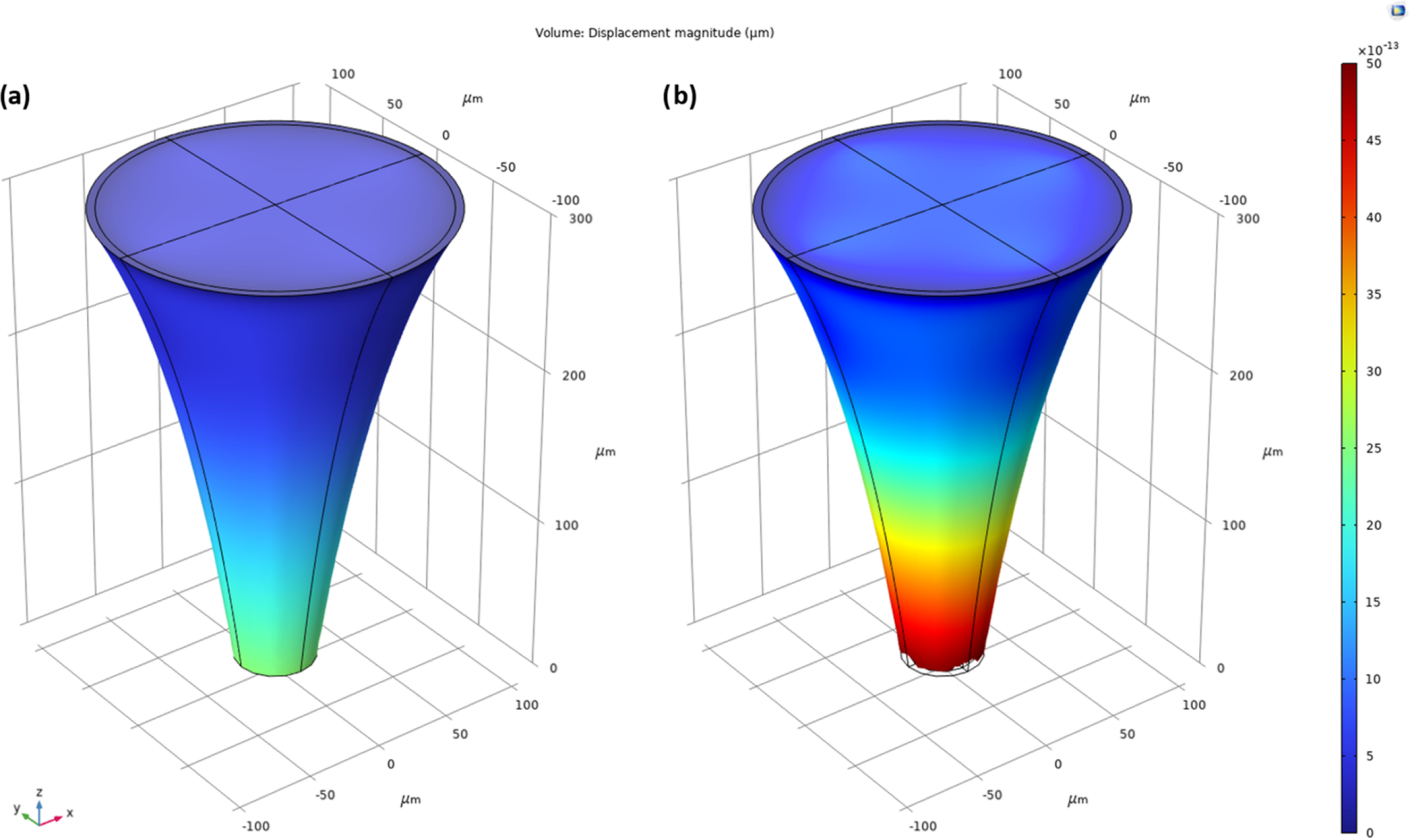
Displacement magnitudes shown in COMSOL for skin forces of (a) a male, aged between 20 and 30 (1.47 × 10^–9^ Pa) and (b) a female, aged between 60 and 70 (2.94 × 10^–9^ Pa). The MN material is stainless steel. Average volumetric flow rates are 1.026 × 10^9^ and 4.213 × 10^9^ μm^3^ s^–1^, respectively.

Finite element analysis is performed on COMSOL Multiphysics by the parametric sweeping method. All the combinations of the chosen model parameters are fed as an input to the Stationary Solver and the outcome was examined both individually and as a data set. Figure 4 shows the simulation results for two cases, where each numeric parameter value is at its minima or maxima, respectively. Solid MN was subjected to compression by skin force, resulting in a slight deformation. Although the MN in Figure 4b underwent more deformation, overall average VFR was higher. Depending on the parameter values, MN deformation did not significantly affect the overall efficiency. The initial design of the MN was used to compare the effects of the skin type and material of the MN (Figure 5). The results have shown that generic materials that were used for MN fabrication have shown similar mechanical behavior, meaning that under the same skin force, deformation of the MN remains constant. VFR within the MN was derived from simulation results for each case using the Derived Values feature in the COMSOL environment and the table containing each parametric value and their respective VFR was exported as text files.

### 3.2 ML Performance Evaluation

Table 4 shows MSE values and R-2 scores of the ML methods. MSE measures the average squared difference between the predicted and actual values, with lower values indicating better performance. A lower MSE indicates that the model is better at predicting the target value, and that the predictions are closer to the true values. In other words, MSE refers to the empirical risk of the predictions. R-2 is another common performance metric used in regression problems, and measures how well the model fits the data compared to a simple baseline model. R-2 values range from 0 to 1, with higher values indicating better performance. An R-2 score of 1 means that the model perfectly fits the data, while an R-2 score of 0 means that the model performs no better than a simple mean model. R-2 score is an indicator of possible overfitting or underfitting issues, although the evaluation of the R-2 score is oversensitive to outliers and insensitive to the difference between predicted outcomes and actual values, thus may not represent the true performance of a model.^34^ These metrics explain how well a model is performing at making predictions and it is useful for comparing models with each other. When a machine learning model has good performance metrics, it means that it is able to make accurate predictions or classifications on the given data set. This is important because it means that the model is more likely to be useful for real-world applications, where accurate predictions are crucial for making informed decisions. Performance is evaluated based on the MSE value.

**Table 4:** Performance Evaluation for Regression Learners

regression learner	hyperparameter optimizer	mean squared error	R-2 score
linear regression	N/A	7.6082 × 10^–3^	0.7414
regression ensemble	Bayesian	4.541 × 10^–4^	0.9778
	none	6.4385 × 10^–4^	0.9733
decision tree regression	Bayesian	3.2133 × 10^–6^	0.9999
	none	4.3767 × 10^–6^	0.9999
support vector regression	Bayesian	9.0913 × 10^–3^	0.7368
	none	8.7686 × 10^–3^	0.7350
generalized additive model	Bayesian	1.7854 × 10^–3^	0.9395
	none	6.9257 × 10^–3^	0.7629
Gaussian process regression	Bayesian	3.2173 × 10^–5^	0.9916
	none	6.3276 × 10^–4^	0.9787
neural network regression	Bayesian	1.5837 × 10^–4^	0.9989
	none	1.2084 × 10^–3^	0.9589

Bayesian hyperparameter optimization resulted in higher performance metrics in most of the methods, with the exception of SVR and NNR models. This may be due to an underestimation of uncertainties or small sample size for a multi-dimensional data set. Table 5 shows the best estimated feasible hyperparameter points for each ML method.

**Table 5:** Best Estimated Feasible Points for Bayesian Hyperparameter Optimization for Each Regression Method

regression learner	hyperparameters	best estimated feasible point
regression ensemble	method	least-square boosting
	number of learning cycles	417
	learning rate	0.65693
	minimum leaf size	12
decision tree regression	minimum leaf size	3
support vector regression	box constraint	34.114
	Kernel scale	3.2839
	margin of tolerance (ε)	0.040221
generalized additive model	initial learning rate for predictors	0.80353
	number of trees per predictor	13
	interactions	9
	initial learn rate for interactions	0.7009
	number of trees per interaction	12
Gaussian process regression	noise standard deviation (σ)	0.00017704
neural network regression	activations	rectifier linear unit
	standardize	false
	regularization rate (λ)	5.6375 × 10^–6^
	layer sizes	[10 272 224]

**Figure 6:**
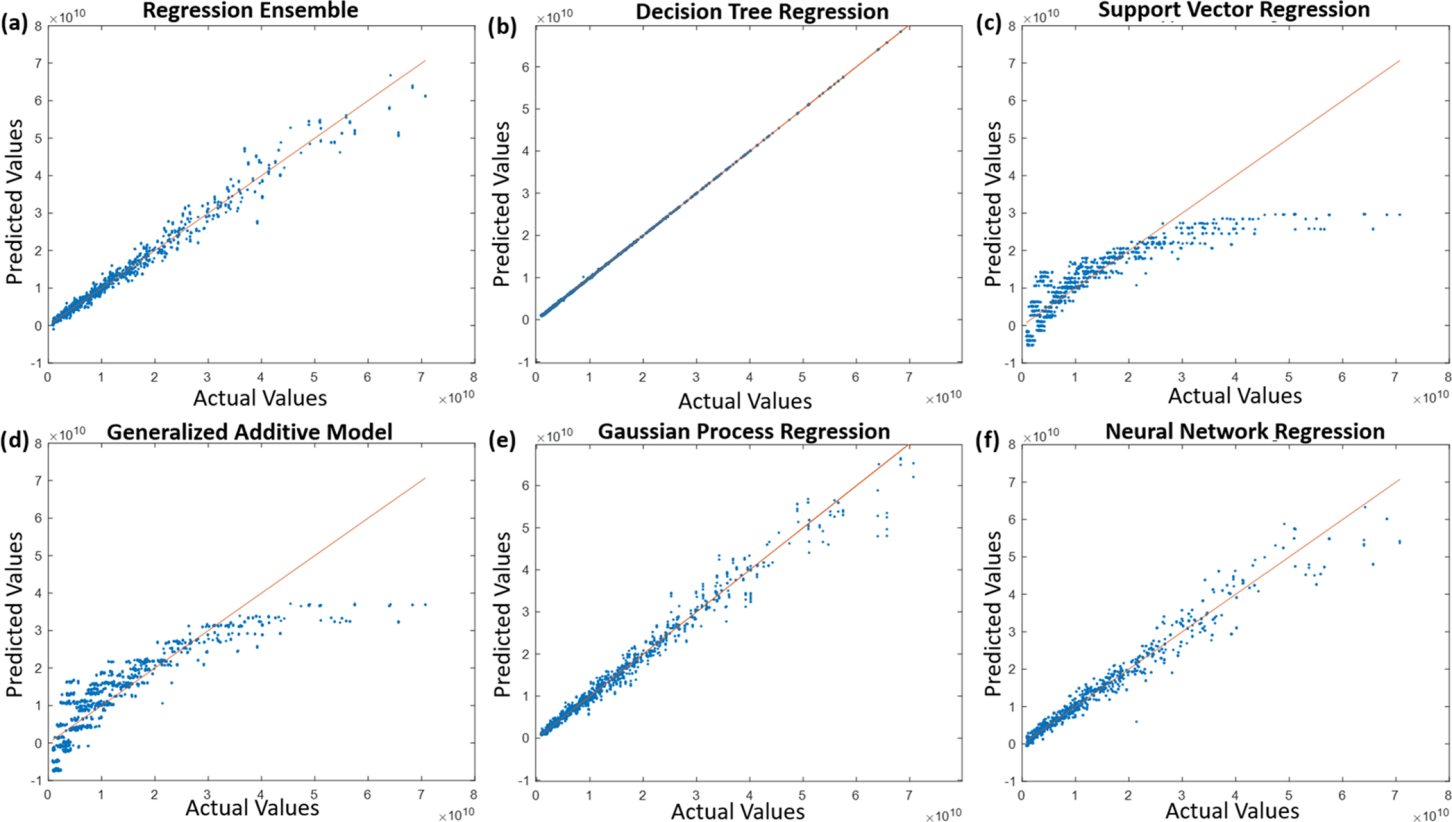
Actual values versus predicted values on testing data set after training (a) regression ensemble (RE), (b) decision tree regression (DTR) model, (c) support vector regression (SVR) model, (d) generalized additive model (GAM), (e) Gaussian process regression (GPR), and (f) neural network regression (NNR) model with the training data set without hyperparameter optimization.

**Figure 7:**
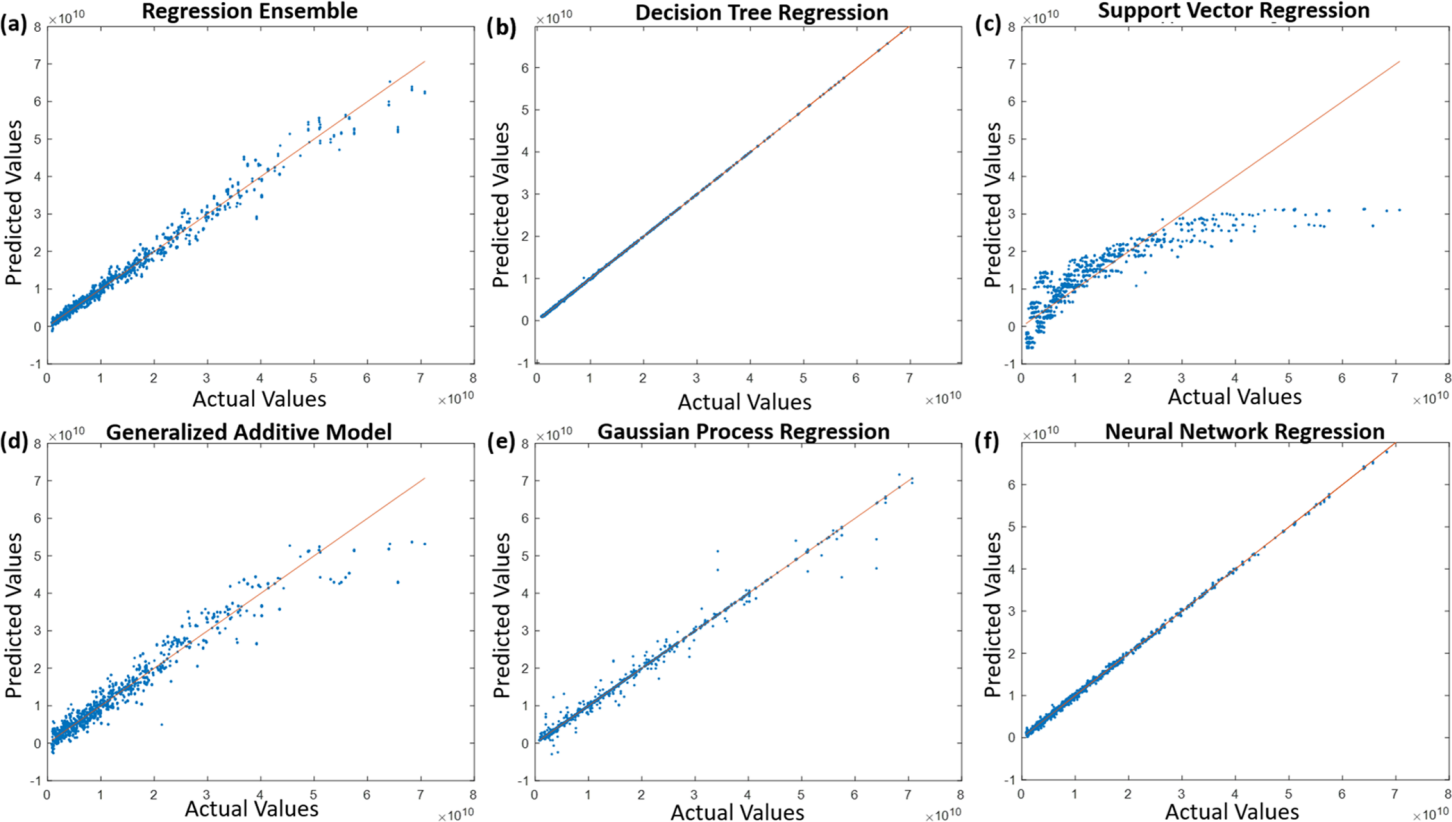
Actual values versus predicted values on testing data set after training (a) regression ensemble (RE), (b) decision tree regression (DTR) model, (c) support vector regression (SVR) model, (d) generalized additive model (GAM), (e) Gaussian process regression (GPR), and (f) neural network regression (NNR) model with the training data set with Bayesian hyperparameter optimization.

The LR model showed poor model performance in comparison to other ML methods (Figure S1 and Table 4). Although certain model parameters had linear relationships with the target variable, overall problem that was defined was not linear. Figures 6 and 7 illustrate the model performance over testing data for the remaining models, where a diagonal line represents the ideal model for which the predicted value is equal to the actual value. The data points that are scattered further away from the line indicates poor model performance. Although SVR models are previously reported to perform better that LR models,^35^ SVR and LR models have shown similar MSE values, 7.6082 × 10^–3^ and 9.0913 × 10^–3^, respectively. Deficient performance of SVR is hypothetical due to a limited number of iterations in hyperparameter optimization. GAM also yields similar MSE values, both with and without hyperparameter optimization, but has a higher R-2 score. RE, GPR, and NNR models yield lower MSE values compared to LR, SVR, and GAM models; with even higher R-2 scores. Finally, DTR is determined to be the best-performing method amongst all investigated ML methods for MN design optimization, with the lowest MSE value of 3.2133 × 10^–6^ and the highest R-2 score of 0.9999.

### 3.3 Graphical User Interface (GUI)

Manually optimizing microneedle parameters can be a time-consuming and challenging task. With the help of a GUI, researchers can easily input their initial parameters, and the system can automatically generate the optimal values for the remaining parameters, saving them a significant amount of time and effort. Additionally, a GUI can provide more accurate results compared to manual design optimization.

**Figure 8:**
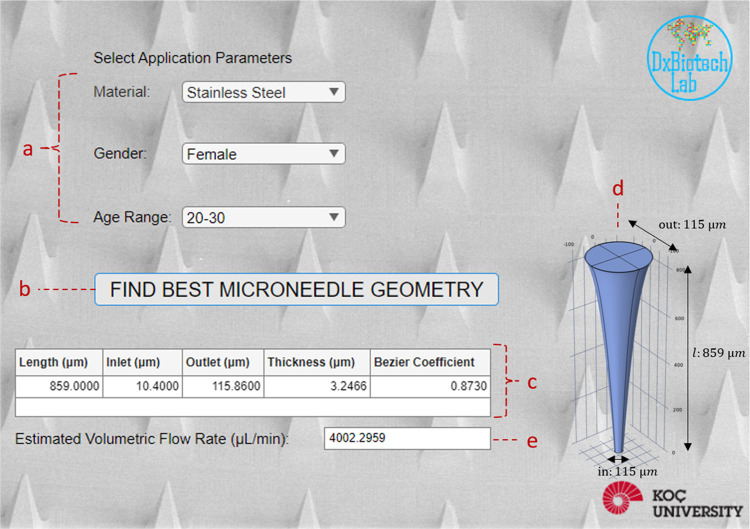
GUI developed for optimal geometric features of MN. (a) User can select the material of the MN and the age and gender of the person that the MN application is targeted for. (b) After selection of the pre-determined parameters, the user can initiate the predictor model to find the optimal geometrical features and estimated volumetric flow rates. After clicking on the button, (c) determined optimal features and (d) visual representation of the MN can be seen in the table. (e) GUI shows the estimated volumetric flow rates in microliters per second.

To facilitate user experience, a MN design optimizing application was developed in the form of GUI with MATLAB App Designer (Figure 8). The aim of the GUI is to enable the user to easily test different combinations of pre-determined variables (MN material, age, and gender) and to obtain the best geometrical features determined for the maximum VFR, which can also be seen on the interface. GUI determines the best parameters by evaluating the results of DT (which yields the best results) model with a random set of initial parameters and iteratively improves the values of parameters for the highest VFR for 30 iterations. Increasing the number of iterations can improve the accuracy but also requires higher processing capability, and therefore increases the processing time.

## 4 Conclusions

This work presented a data-driven approach to the design optimization of MNs where a data set containing numerous different sizes and shapes of microneedles was generated via COMSOL parametric sweep. The data set is utilized to fit a ML model that can predict VFR of the fluid inside a MN for any given set of parameters. This methodology aims to eliminate the disadvantages of the trial-and-error method and accordingly the human factor from design optimization problems, while enabling investigation of large sets of case studies that otherwise would be difficult to obtain. Further studies can be conducted with larger sample sizes, different parameters, or a different problem definition such as cost optimization or fabrication feasibility. The results can be reproduced for any material, but appropriate conditions should be introduced to the model accordingly in order to meet the fabrication limitations. ML methods can be improved by optimizing the hyperparameters with more iterations. Aside from the improvements on ML methods, preprocessing and structure of the data are crucial to predict accuracy. Larger training data sets and cross-validation can advance predictor performance. This method can also be implemented with different critical features of MN and can be evaluated for different target parameters such as cost, pain, and fabrication feasibility. Additionally, this ML approach of action can be implemented in biomedical instruments, including wearable devices and drug delivery systems.

## Supporting Information Available

Performance evaluation for linear regression (LR) model on training data set (PDF).

## Abbreviations

CFD: computational fluid dynamics
DTR: decision tree regression
FEM: finite element method
GAM: generalized additive model
GPR: Gaussian process regression
GUI: Graphical user interface
ISF: interstitial fluid
LR: linear regression
ML: machine learning
MN: microneedle
MSE: mean squared error
NLP: nonlinear programming
NN: neural network
NNR: neural network regression
QBC: quadratic Bezier curve
RE: regression ensemble
SVM: support vector machine
SVR: support vector regression
VFR: volumetric flow rate
