# Supplementary material for: Machine Learning-Enabled Optimization of Interstitial Fluid Collection via a Sweeping Microneedle Design
Source: ACS Omega. 2023 May 31;8(23):20968–78. doi: 10.1021/acsomega.3c01744 (PMC10268608; doi:10.1021/acsomega.3c01744)
Supplement: Supplementary file 1 — ao3c01744_si_001.pdf [file ao3c01744_si_001.pdf]

Supplementary Information

Machine Learning-enabled optimization of interstitial fluid collection via sweeping microneedle design

Ceren Tarar[1], Erdal Aydın[2,3], Ali K. Yetisen[4], and Savas Tasoglu[5,6,7,8,9,10,¥]

[1]Department of Biomedical Sciences and Engineering, Koç University, Sariyer, Istanbul, Turkiye 34450.
[2]Department of Chemical and Biological Engineering, Koç University, Sariyer, Istanbul, Turkiye 34450.
[3]TUPRAS Energy Center (KUTEM), Koç University, Istanbul 34450, Turkey
[4]Department of Chemical Engineering, Imperial College London, London SW7 2AZ, UK
[5]Koc University Is Bank Artificial Intelligence Lab (KUIS AILab), Koç University, Sariyer, Istanbul 34450, Turkiye
[6]Koç University Translational Medicine Research Center (KUTTAM), Koç University, Istanbul, Turkey 34450

[7]Boğaziçi Institute of Biomedical Engineering, Boğaziçi University, Çengelköy, Istanbul, Turkiye 34684.
[8]Department of Mechanical Engineering, Koç University, Sariyer, Istanbul, Turkiye 34450.
[9]Koç University Arçelik Research Center for Creative Industries (KUAR), Koç University, Sariyer, Istanbul, Turkiye 34450.

[¥]Corresponding Author: Savas Tasoglu (stasoglu@ku.edu.tr)

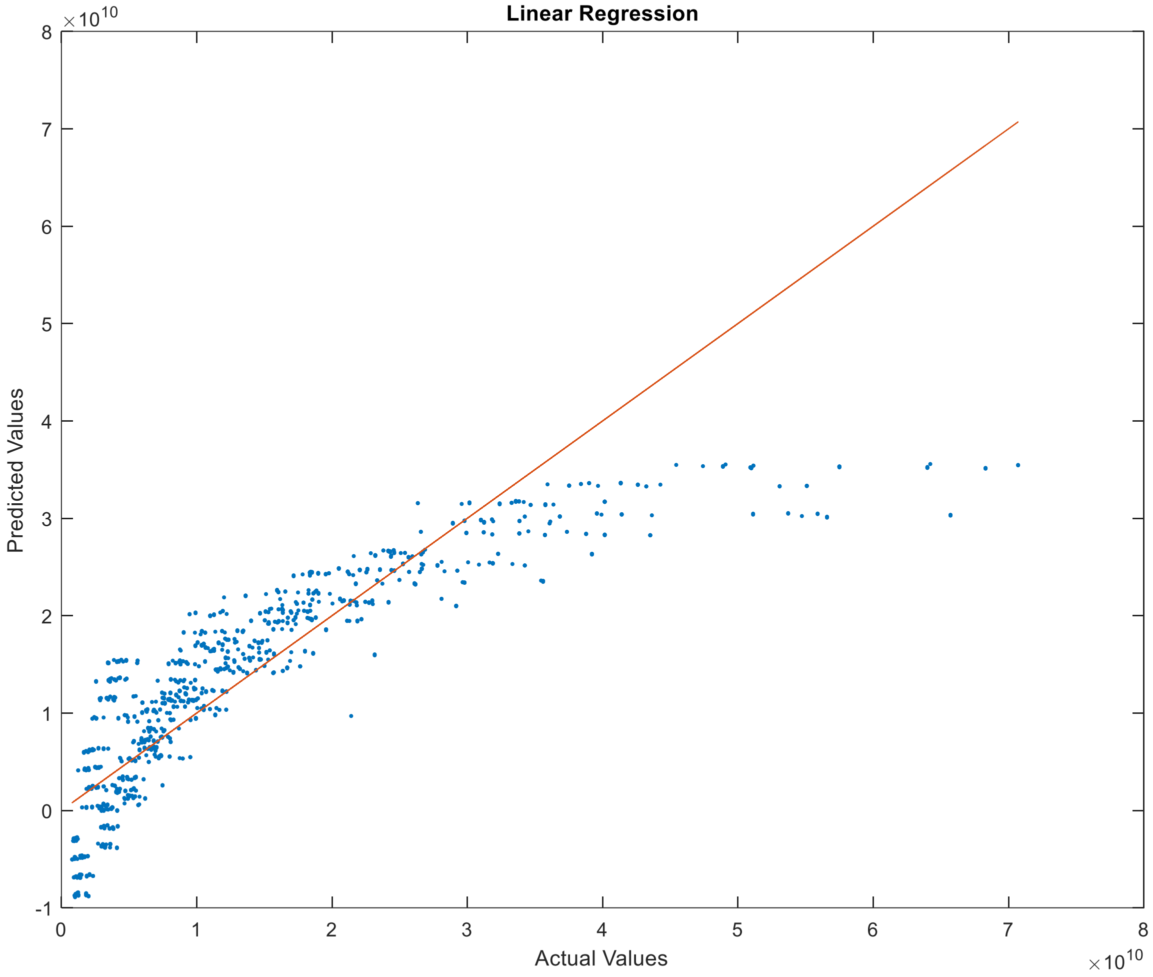

Figure S1.: *The plot of actual values versus predicted values on testing dataset after training linear regression (LR) model with the training dataset. Trained LR model evaluation has the MSE error of 7.6082e-03 and R-2 score of 0.7414.*
